# Room temperature excitation spectroscopy of single quantum dots

**DOI:** 10.3762/bjnano.2.56

**Published:** 2011-08-30

**Authors:** Christian Blum, Frank Schleifenbaum, Martijn Stopel, Sébastien Peter, Marcus Sackrow, Vinod Subramaniam, Alfred J Meixner

**Affiliations:** 1 Nanobiophysics Group and MESA+ Institute for Nanotechnology, University of Twente, PO Box 217, 7500 AE Enschede, The Netherlands; 2 Center for Plant Molecular Biology, Biophysical Chemistry, University of Tübingen, Auf der Morgenstelle 18, 72076 Tübingen, Germany; 3 Institut für Physikalische und Theoretische Chemie, University of Tübingen, Auf der Morgenstelle 8, 72076 Tübingen, Germany; 4 present address: Picoquant GmbH, Rudower Chaussee 29, 12489 Berlin, Germany

**Keywords:** blinking, excitation spectrum, quantum dots, single molecule spectroscopy, supercontinuum laser

## Abstract

We report a single molecule detection scheme to investigate excitation spectra of single emitters at room temperature. We demonstrate the potential of single emitter photoluminescence excitation spectroscopy by recording excitation spectra of single CdSe nanocrystals over a wide spectral range of 100 nm. The spectra exhibit emission intermittency, characteristic of single emitters. We observe large variations in the spectra close to the band edge, which represent the individual heterogeneity of the observed quantum dots. We also find specific excitation wavelengths for which the single quantum dots analyzed show an increased propensity for a transition to a long-lived dark state. We expect that the additional capability of recording excitation spectra at room temperature from single emitters will enable insights into the photophysics of emitters that so far have remained inaccessible.

## Introduction

Since the first demonstration of single molecule fluorescence spectroscopy over two decades ago, techniques to detect and characterize the emission from single emitters have become increasingly sophisticated and versatile. These developments have made optical single molecule spectroscopy an indispensable tool to address complex problems in chemistry [1–3], in material sciences [4–6], and in life sciences [7–11].

A number of parameters that characterize single molecule emission are now routinely accessible at ambient temperatures, including emission intensity and polarization [12–13], fluorescence lifetime [14–16], and the emission spectrum [17–19]. Access to these parameters yields unique insights into distinct properties of single molecules, and enables the determination of the distributions of the relevant experimental parameters, revealing, for example, distinct sub-states and energetic levels in a heterogeneous population [20–21]. Furthermore, external tailoring or directing of molecular emission has also been reported [22–24].

However, a detailed study of frequency resolved excitation dependent processes at the single molecule level, at room temperature, has not been experimentally achievable so far. Appreciation of these processes is fundamentally important for the understanding of the basic physics and for applications in next-generation photonic devices. The primary challenge has been the intrinsic difficulty in measuring the absorption of a single emitter at room temperature due to the extremely low signal to noise ratio. Although recent reports have demonstrated the detection of single molecule absorbance [25–27], a complete single molecule absorbance spectrum at room temperature has not yet been reported. A complementary approach to access the frequency dependent coupling of an emitter to an external electromagnetic field is based on photoluminescence excitation spectroscopy. Single emitter photoluminescence excitation microscopy has been already achieved in the early days of single molecule detection, but has been limited to experiments at cryogenic temperatures where the linewidths of individual emitters are not inhomogeneously broadened [28–29]. Hence, only a very limited excitation wavelength range was required to resolve individual absorbance properties of a single emitter at low temperatures.

In this paper, we describe the first successful acquisition of single emitter excitation spectra under ambient conditions over a wide spectral range. We combine a tunable white-light laser source with a confocal microscope with single molecule detection sensitivity to demonstrate excitation spectra of isolated semiconductor nanocrystals. These fluorophores, often referred to as quantum dots, have unique optical properties [30–35], including a narrow and tailored luminescence emission spectrum and significantly enhanced photostability compared to organic fluorophores. These properties make quantum dots promising nanomaterials in various fields of research, ranging from in vivo probes in the life-sciences [10,36–37] to single photon light sources in telecommunications [38] or quantum computing [30,35]. We demonstrate here how single emitter excitation spectroscopy provides a valuable addition to the range of single emitter spectroscopy techniques, yielding new insights into the complex photophysics of quantum dots.

The excitation spectrum, commonly used in ensemble fluorescence spectroscopy, depicts the evolution of the emission intensity recorded in a fixed spectral detection window upon scanning the excitation wavelength. Moreover, the excitation spectrum of an emitter coincides with its absorbance spectrum if the quantum efficiency is independent of excitation wavelength, which is generally assumed to be true for most emitters over large wavelength ranges. Hence, measurement of the excitation spectra of individual quantum dots permits access to the individual absorbance properties that are not accessible by common single molecule techniques or not at all by ensemble approaches.

## Results and Discussion

**Figure 1:**
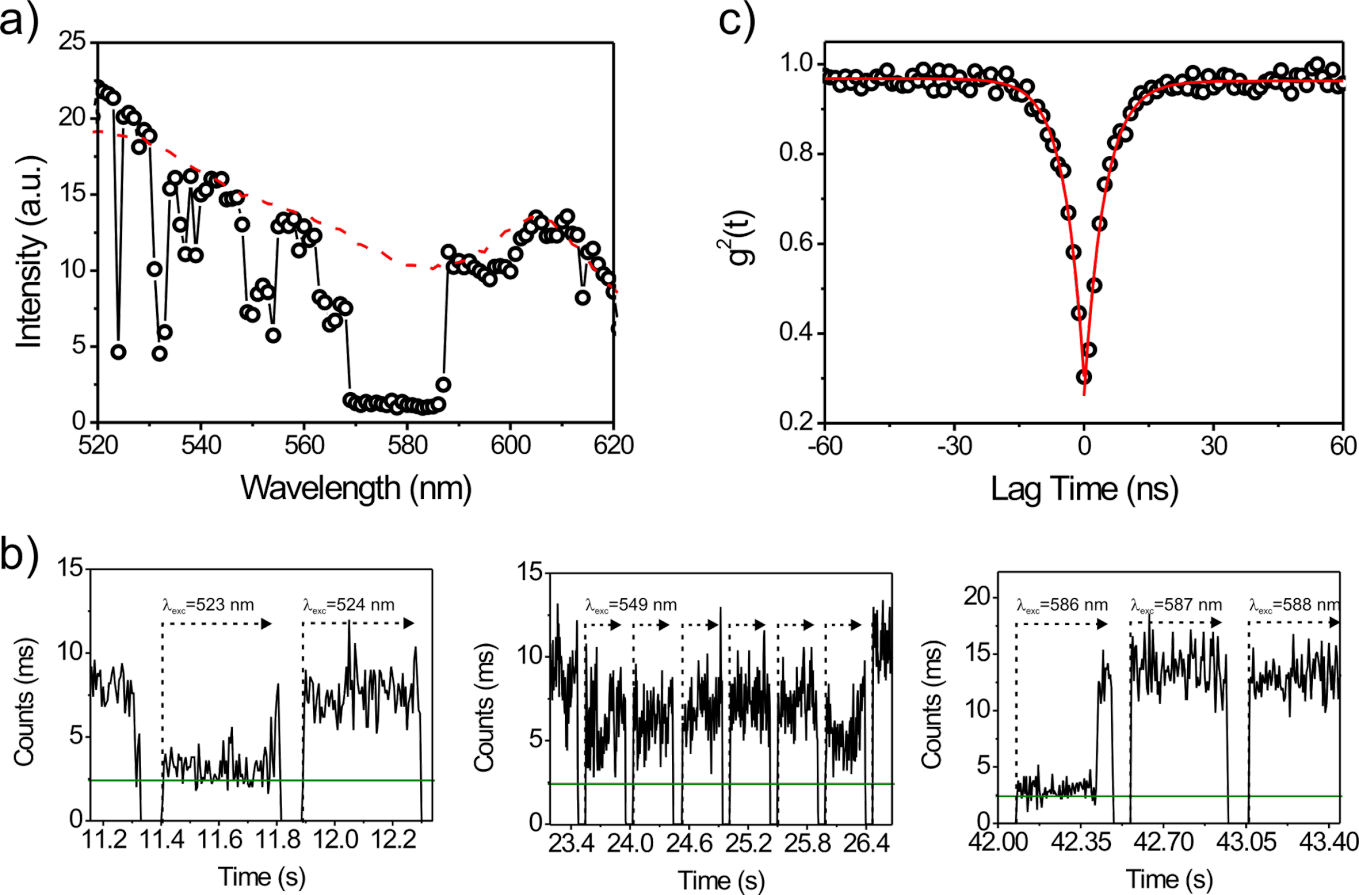
Single emitter characteristics observed by excitation spectroscopy of isolated quantum dots. a) Excitation spectrum of a single quantum dot (open circles) with typical intensity intermittencies that result in drops and gaps in the spectrum. The ensemble excitation spectrum is shown for comparison (red dashed line). b) Intensity trajectories of a single quantum dot for selected excitation wavelengths (green line marks the background level). c) Photon antibunching curve of a single quantum dot.

In this study, we recorded excitation spectra of 48 individual CdSe/ZnS core–shell quantum dots at room temperature. Since the occurrence of emission intermittencies (blinking) is a clear indication for the observation of a single emitter, and because blinking of quantum dots is still not fully understood, we did not apply any measures to suppress or minimize blinking. The single quantum dot excitation spectra recorded exhibited the main characteristics of a declining slope from shorter to longer wavelengths, and a peak close to the band edge transition, which we identify as the 1S(e)-2S_3/2_(h) transition [39]. However, we find distinct differences in the individual spectra that can be attributed to individual photophysical properties of the analyzed single quantum dots as well as to the well-known transitions of single emitters to dark, non-emitting, states. A typical example of an excitation spectrum obtained from a single quantum dot is shown in Figure 1a. In contrast to the ensemble spectrum, we observed distinct dips and gaps in the single quantum dot excitation spectra, which in principle could either result from blinking or reflect the photophysical properties of the quantum dot.

Semiconductor quantum dots exhibit a discrete structure of quantized energy states. Hence, one would expect to observe discrete bands in both the excitation and absorbance spectra when the excitation wavelength is in resonance with a transition to such a discrete state. Low temperature experiments showed narrow emission lines [40], but also revealed that only a few sharp transitions in the direct vicinity of the band edge can be found, while at higher excitation energies the optical transitions merge into a dense quasicontinuum [41]. At room temperature, these sharp transitions experience inhomogeneous broadening effects, mainly due to lattice vibrations. It is therefore not surprising that no sharp transitions are resolved in the room temperature ensemble excitation spectra of quantum dots.

The spectra demonstrate obvious intensity fluctuations of different magnitudes within a single measurement interval, which are characteristic of the emission from a single emitter. These fluctuations have been reported for semiconductor quantum dots, and have only recently been circumvented in exceptional cases [42–44]. The intensity blinking of the quantum dot can be visualized from the intensity trajectories that were recorded with a temporal resolution of 5 ms (Figure 1b). The breaks to true zero between trajectories are instrument-related, and indicate the change of the excitation wavelength, while the green line marks the background signal level without quantum dot emission, attributed to the emitter being trapped in a dark state.

Clearly, these drops to the background level are not related to narrow absorbance lines due to the band structure of the semiconductor quantum dots. In this case, drops in the recorded intensity would result in excitation wavelengths for which no emission can be recorded. The start and end of such a dark interval would then have to coincide with the start of a new excitation wavelength recording interval. We did not observe this behavior and the beginning and end of a dark period occurred stochastically.

The left panel of Figure 1b depicts the intensity evolution for excitation from 522–524 nm. For these wavelengths, the excitation spectrum showed strong intensity fluctuations. At λ_ex_ = 522 nm, an intensity jump was observed immediately before the subsequent wavelength change. For λ_ex_ = 523 nm, the quantum dot was still in a non-emitting off-state, indicated by the signal intensity being at the background level. After 11.8 s it returned into a stable on-state after some initial short "bursts". Accordingly, transitions to short lasting off-states resulted in sudden dips in the excitation spectrum, and the intensity in the excitation spectrum did not drop to the background level, as the quantum dot was not dark during the whole integration interval.

The middle panel in Figure 1b depicts a decreased emission intensity that varied over time but did not drop to the background level. These variations in the observed emission intensity can be explained either as the result of fast blinking, below the time resolution of the experiment, or by transitions of the quantum dot to a dim (weakly-emitting) state [45]. As a result of this reduced emission we observed drops in the excitation spectrum over a number of wavelengths as can be seen around 550 nm in Figure 1a. Finally, we often found extended gaps in the excitation spectrum of single quantum dots as can be seen in the spectrum between 569 nm and 587 nm (Figure 1a). This gap in the excitation spectrum is attributed to a long lasting dark state followed by the return to an emitting state as can be seen in the right panel of Figure 1b. It is important to note that the observed drops in intensity do not correlate with changes in the excitation wavelength. Clearly, the observed drops and gaps in the spectrum would vanish if exclusively emitting states were sampled for each excitation wavelength. For detection intervals where a transition to or from a dark state occurred (e.g., Figure 1b, right panel) this correction can easily be performed by considering just the higher emission intensity level. Drops and gaps in the spectrum originating from dim states or very fast transitions below the sampling resolution, and from long lasting dark states longer than the integration time per excitation wavelength, could be avoided by repeated, possibly faster, scanning of the excitation wavelength, which requires further technical development for implementation in future studies.

We argue that the observed transitions between on- and off-states reflect the intrinsic emission characteristics inherent to individual quantum dots. In addition to the intensity blinking observed, we further confirmed that we were addressing a single emitter and thus single photons by analyzing the coincidence of detected photons in time using a Hanbury Brown and Twiss configuration [46]. The resulting photon-antibunching curves recorded in this manner exhibited a near zero correlation (g^2^ = 0.3) for the detection of two photons at the same time, as shown for a typical example in Figure 1c. Photon-antibunching data give strong evidence for the observation of single emitters [47], since a single emitting system intrinsically cannot emit two photons at the same time. Generally, g^2^ values below 0.5 are accepted as a proof of single molecule observation [34].

**Figure 2:**
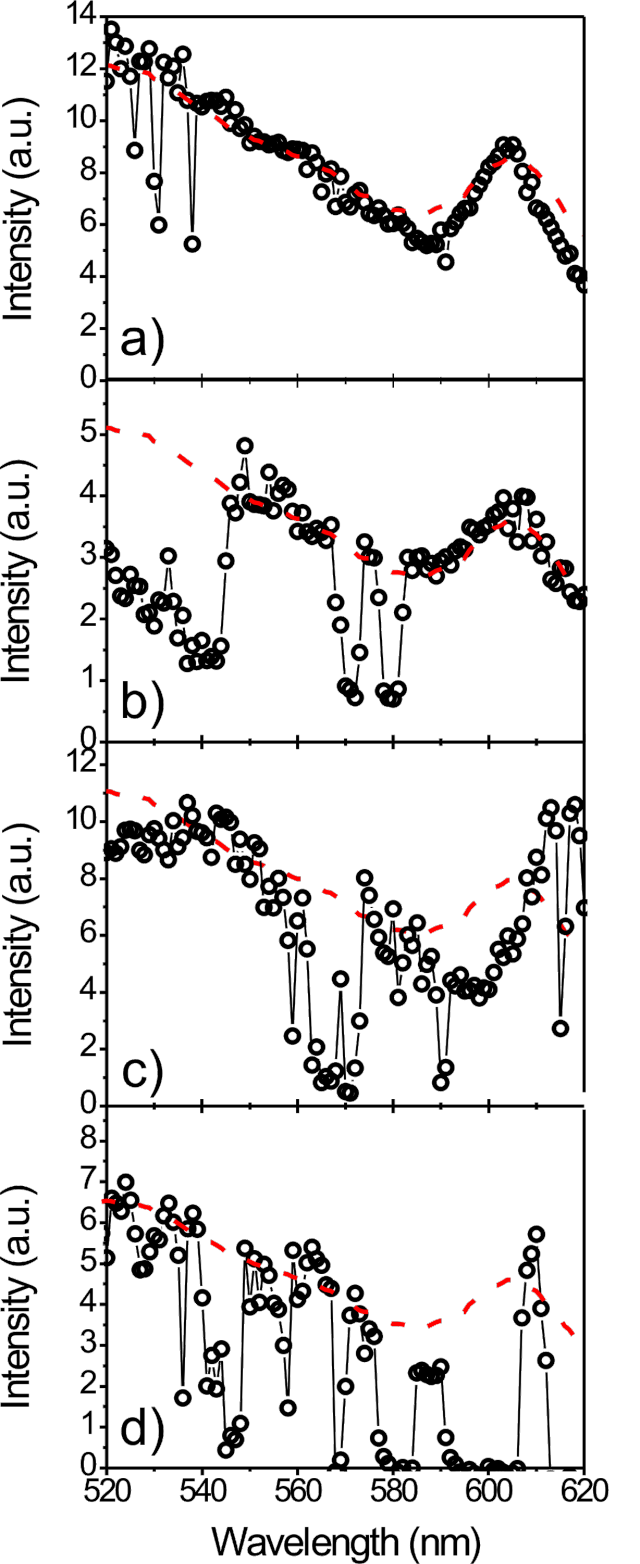
Single QD photoluminescence excitation spectra. For comparison, the ensemble excitation spectrum is shown as the red dashed line. The spectra show varying degrees of emission intermittencies visible as drops and gaps in the spectra. Especially in the wavelength region of the pronounced 1S(e)-2S_3/2_(h) transition the spectra show distinct differences between different quantum dots, reflecting the individual nature of each quantum dot.

The recorded spectra show varying degrees of blinking, ranging from spectra exhibiting almost no dips and gaps due to emission intermittencies (Figure 2a), to spectra where numerous transitions between emitting and dark states can be observed (Figure 2b–d). The excitation spectrum shown in Figure 2a is very intense and shows only minor signs of blinking, and is based on the detection of ~2·10^5^ photons emitted from the sampled quantum dot. This number of detected photons is comparable to the average number of photons that can be detected from organic fluorophores [48–49] and suggests that single emitter excitation spectroscopy could also be used for classes of emitters other than the very photostable quantum dots analyzed in this study.

**Figure 3:**
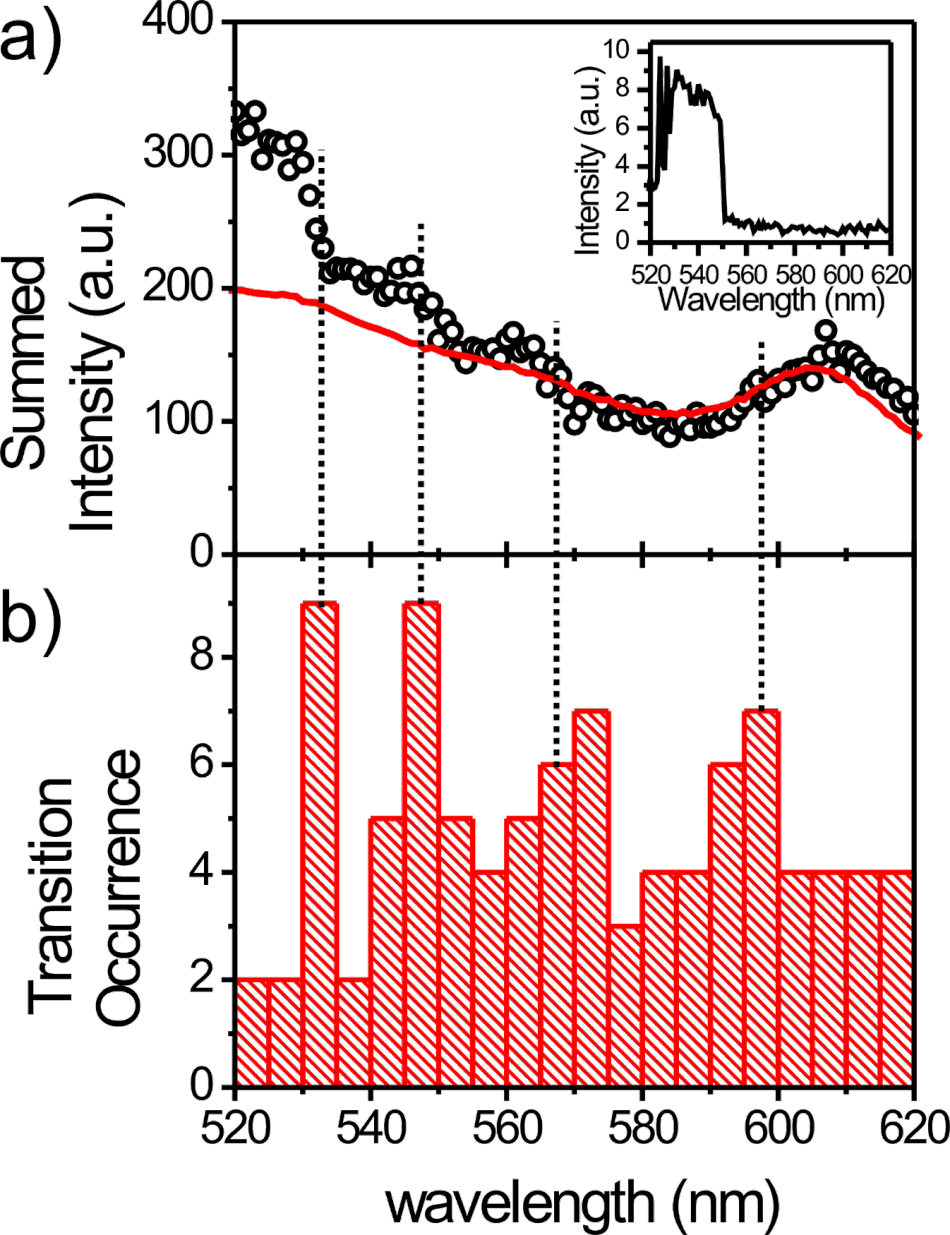
Single quantum dot excitation spectra reveal distinct excitation wavelengths with increased probability for a dark-state transition. a) sum spectrum of 48 excitation spectra of individual quantum dots (circles). Red: Ensemble excitation spectrum. Inset: Single quantum dot excitation spectra undergoing a transition to a dark state. b) Histogram of transitions to a long lived dark-state.

The recorded data further enables the detailed analysis of the influence of the excitation wavelength on the blinking of single quantum dots. Numerous studies on single quantum dots have shown complicated luminescence intermittency, or blinking, with power law statistics over many decades in time. In most of these studies a single excitation wavelength was used, and only recently has the first in-depth investigation of quantum dot blinking comparing a small number of different excitation wavelengths been published [32]. These studies were based on the statistical analysis of different quantum dots and an analysis of the emission of individual emitters using different excitation wavelengths has not yet been achieved. The approach presented here makes it possible to study the response of individual emitters to changes in excitation wavelength over a broad range. Hence, increased photoluminescence intermittency for certain excitation wavelengths will result in systematically reduced emission intensity for this wavelength in our study. We therefore calculated the sum of the photoluminescence excitation spectra from all single quantum dots analyzed in our study and compared it to the ensemble spectrum recorded with a calibrated ensemble spectrometer (Figure 3a). The summed photoluminescence excitation spectrum shows a number of interesting characteristics. Globally, blinking that was visible in the individual excitation spectra averages out, and the sum spectrum shows no explicit gaps where the intensity suddenly drops and then recurs. However, there are clear differences between the ensemble photoluminescence excitation spectrum and the summed excitation spectra from single quantum dots. On the short wavelength side, the sum spectrum declines much faster than the ensemble spectrum, while on the long wavelength side there is good agreement between the positions of the pronounced 1S(e)-2S_3/2_(h) transition. This discrepancy on the short wavelength side of the sum spectrum is not apparent in the individual spectra and can be understood from the details of how each single emitter excitation spectrum was recorded, that is, by considering that the excitation wavelength was always scanned from short to long wavelength. As the transition to dark states is driven by the excitation light, the probability to find a single quantum dot in a non-emitting state is minimal at the start of the experiment. Over time, which translates to longer excitation wavelengths in our experiment, the probability to find a quantum dot in a long-lived dark state increases. In the extreme case, a transition to a dark state occurs and photoluminescence is not regained before the end of the experiment (Figure 3a, inset). Thus, shorter excitation wavelengths are overweighted in the sum spectrum in the excitation scheme used, which was dictated by the monochromator used for these experiments (see Supporting Information File 1 for details) that only allows for scanning of the excitation from low to high wavelengths. One promising way to overcome this limitation in future experiments is to use an acousto-optical tunable filter (AOTF) for fast wavelength selection and bidirectional wavelength scanning.

However, not all discrepancies between the sum spectrum and the ensemble spectrum can be explained by the details of the excitation scheme used. If the probability for a transition to a long-lived dark state is independent of the excitation wavelength, a steady decline of the summed single quantum dot excitation spectrum approaching the spectral shape of the ensemble spectrum is expected when both spectra are normalized to the long wavelength edges of the spectra. Indeed, we find good agreement between the sum and ensemble spectra for excitation wavelengths beyond ~580 nm, which suggests only a minor influence from transitions to dark states that do not recover during the entire data acquisition time in this wavelength range. On the other hand we find large deviations between the sum and ensemble spectra for excitation wavelengths below ~580 nm. This observation is consistent with reports that excitation in the band gap area results in little blinking compared to excitation above the band gap [32]. However, we do not see a smooth decline of the excitation sum spectrum, but observe what appear to be a number of steps (~530 nm, ~550 nm, 565 nm, and 595 nm (less prominent); Figure 3a). These drops in the sum spectrum indicate that at the associated excitation wavelengths an increased probability of an intensity drop, that is, of a transition to a dark state, exists. The drops at 530 nm, 550 nm and 570 nm appear to be weakly reflected in the bulk spectrum. Since transitions to dark states are not sampled in the bulk spectrum due to the comparatively low excitation powers used to record bulk spectra, these similarities might point towards the molecular mechanism underlying the increased probability for a transition to a dark state. To verify that the observed steps in the sum spectrum indeed result from an increased number of single quantum dots changing to a dark state, we created a histogram of the wavelengths for which a transition to a dark state could be observed (Figure 3b). To estimate the statistical significance of the distribution obtained we determined the p-value assuming equal probability of a dark state transition for all power normalized excitation wavelengths. We obtain a p-value of 0.06 indicating a statistically significant result since there is only a low probability of ~6% that the observed distribution originates from a random distribution of dark state transitions. The histogram shows significant peaks in the frequency of a dark state transition for the excitation wavelengths ~532 nm, ~548 nm, ~570 nm and ~595 nm. All four peaks are correlated to a signature in the sum spectrum.

The data suggest that there is not only a large difference in the probability of a transition to a dark state for excitation in the band gap compared to excitation above the band gap, but that there are additionally certain excitation wavelengths which preferentially induce transitions to dark states. We exclude the idea of increased blinking rates for lower wavelengths due to increased absorbance and thereby a higher probability of an Auger assisted ionization, since the excitation powers used were smaller at lower wavelengths than at higher wavelengths. Additionally such a mechanism cannot explain the increased probability of a dark state transition for certain wavelengths only. Our data indicates that the formation of dark states shows a complex dependence on the excitation wavelengths used, suggesting that dark states can be reached via different pathways that can be accessed preferentially by using certain excitation wavelengths. Besides details on the wavelength dependent blinking of single emitters, our data also give access to the individual spectral properties of the quantum dots. In Figure 2 we show some typical examples of single quantum dot excitation spectra. As a guide to the eye and for comparison, the reference ensemble excitation spectrum is plotted as a dashed red line in each panel of Figure 2.

Comparing the excitation spectra of different quantum dots we find both striking similarities and some clear differences between the spectra. The single quantum dot spectra are always enveloped by the ensemble spectrum below ~580 nm (Figure 2, Figure 1a). Besides dips and gaps due to the blinking behavior on different timescales, we see no significant differences between the single quantum dots or distinct individual features in this part of the spectrum.

In general, observed wavelength dependent changes in photoluminescence can result from changes in either the absorbance or the photoluminescence quantum yield. Although excitation wavelength dependent changes in photoluminescence quantum efficiency have been discussed [50–51], Tonti et al. were able to show that there is no intrinsic deviation between the excitation and absorbance spectra of quantum dots once all corrections and sample handling are properly performed [52]. This result implies that the photoluminescence quantum yield in CdSe quantum dot ensembles is independent of the excitation wavelength, and that analyzing the excitation spectra also allows one to draw conclusions about the absorbance spectra of single quantum dots. Following from this, the observed behavior directly results from the differences in the absorbance at different wavelengths of the quantum dots. The spectral region below 580 nm, where we find no clear signs of individual spectral behavior from individual quantum dots, is exactly that region where a quasicontinuum of optical transitions was observed at cryogenic temperatures, corresponding to low lying energy barriers between distinct states predicted by the theory. It is therefore not surprising that, except for blinking events, no individual characteristics of the observed quantum dots can be identified at room temperature in this wavelength region.

The picture changes significantly when looking at the pronounced transition at ~605 nm, closer to the band edge, where we find clear differences in the shape, height and spectral position of this peak in the individual excitation spectra. Variations in the spectral position of this peak from individual quantum dots are attributed to differences in the size of the individual quantum dots. As expected for a single molecule study, we also find that the width of the transition is generally smaller than the width of the ensemble transition. This broadening in the ensemble or summed single molecule spectra results from the superposition of a large number of excitation spectra of single quantum dots of varying peak wavelength. Interestingly, we find some excitation spectra that do not show narrowed spectral features, e.g., Figure 2b, pointing towards the existence of a phenomenon equivalent to spectral emission diffusion [53–54] in the excitation spectrum and, by inference, in the absorbance. Especially since we only find minor variations in the excitation spectra at the short wavelength side below 580 nm and do not observe individual fingerprints in this area, the observation of not only the spectral position but also the different relative height and shape for the 1S(e)-2S_3/2_(h) transition reflects the individual characteristics of each single quantum dot. Moreover, our spectra strikingly show that the different heights and shapes of the transitions are not correlated with their spectral position and hence with the particle size, suggesting different molecular origins. The shape and height of the peak from the 1S(e)-2S_3/2_(h) transition is determined by the coupling between these states. It has been shown that transitions close to the band edge show a fine structure splitting into sublevels due to the quantum dot crystal field, shape anisotropy, and confinement enhanced electron-hole exchange interactions [55]. Although considerably broadened at room temperature and hence invisible in the ensemble, the variation of the height and shape of the peak around 605 nm, which we have observed for individual quantum dots, reflects details of these transitions. Since the differences in relative height of spectral features in the photoluminescence excitation spectrum represent the strength of the coupling between the ground and excited state of the individual quantum dots, it is likely that the observed differences in the individual excitation spectra reflect the differences in the photoluminescence quantum efficiency of individual quantum dots, as previously reported [56–57].

## Conclusion

We have recorded for the first time single molecule excitation spectra at room temperature. The required spectrally narrow excitation over a wide spectral range of 100 nm was realized by using a monochromator to select the excitation wavelength from a supercontinuum white light source. The suitability of our approach and its potential were demonstrated by studying single quantum dots. The single emitter nature of our quantum dot samples was confirmed by photon antibunching experiments. Analysis of the single quantum dot excitation spectra gave access to hitherto unexplored details of this class of emitters. For the CdSe nanocrystals investigated, we found strong indications for an increased probability for a transition to long-lived dark states at specific excitation wavelengths, suggesting that these wavelengths are unsuitable for single photon applications. The excitation spectra showed no clear individual features for excitation wavelengths well above the band gap, but exhibited large differences for transitions close to the excitonic peak, representing the fine structure splitting into energetic sublevels of the individual quantum dots. Further, we found variations in the width and the spectral position of transitions for individual quantum dots.

On the basis of these investigations of quantum dots, we expect single molecule excitation spectroscopy to become a valuable addition to the established single molecule spectroscopy methods. The approach will not only aid in the analysis of isolated dyes or nanoparticles but also prove valuable in analyzing complex emitting systems such as Förster resonance energy transfer (FRET) pairs, fluorescent proteins and upconversion particles.

## Experimental

We realized an instrument capable of single molecule excitation spectroscopy by combining a high power supercontinuum white light source (Fianium SC-400pp) with a grating spectrometer (Spectra Pro 300i, Acton Research) for excitation wavelength selection and a custom built confocal scanning stage single molecule detection microscope (for details see Supporting Information File 1). Experiments were carried out in two steps. First, the quantum dots immobilized in a thin polymer layer at low concentration (*c*_QD_ = 5 × 10^−10^ M) were visualized by creating a raster scanned emission intensity image of an 20 × 20 μm^2^ area of the sample using a fixed excitation wavelength (λ_ex_ = 600 nm). After localization, the single quantum dots were positioned in the laser focus and the excitation wavelength was swept from 520 nm to 620 nm in increments of 1 nm. For each excitation wavelength the emitted fluorescence intensity was recorded for 400 ms, followed by an increment in the excitation wavelength during which data acquisition was disabled (~70 ms duration). In this way we obtained 400 ms fluorescence intensity trajectories depicting the evolution of the emission intensity for each excitation wavelength, interspersed with ~70 ms zero-intensity periods representing the excitation wavelength increment (Figure 1). To obtain the fluorescence excitation spectrum, the total emission intensity per excitation wavelength was integrated and calibrated using a reference spectrum recorded for a film of emitters (i.e., an ensemble of emitters) to compensate for wavelength dependent excitation power and detection efficiency (for details see Supporting Information File 1).

## Supporting Information

Supporting Information features detailed description of instrumentation, and data acquisition and correction procedures.

File 1 Experimental details.
