# Supplementary material for: Room temperature excitation spectroscopy of single quantum dots
Source: Beilstein J Nanotechnol. 2011 Aug 30;2:516–24. doi: 10.3762/bjnano.2.56 (PMC3190622; doi:10.3762/bjnano.2.56)
Supplement: File 1 — Experimental details. [file Beilstein_J_Nanotechnol-02-516-s001.pdf]

Supporting Information

for

Room temperature excitation spectroscopy of single quantum dots

Christian Blum[1,§], Frank Schleifenbaum[2,§], Martijn Stopel[1], Sébastien Peter[2], Marcus Sackrow[3,4], Vinod Subramaniam*[1] and Alfred J. Meixner*[3]

Address: [1]Nanobiophysics Group and MESA[+] Institute for Nanotechnology, University of Twente, PO Box 217, 7500 AE Enschede, The Netherlands, [2]Center for Plant Molecular Biology, Biophysical Chemistry, University of Tübingen, Auf der Morgenstelle 18, 72076 Tübingen, Germany, [3]Institut für Physikalische und Theoretische Chemie, University of Tübingen, Auf der Morgenstelle 8, 72076 Tübingen, Germany and [4]present address: Picoquant GmbH, Rudower Chaussee 29, 12489 Berlin, Germany

Email: Alfred J. Meixner* - alfred.meixner@uni-tuebingen.de; Vinod Subramaniam* - v.subramaniam@utwente.nl

* Corresponding author

[§] CB and FS contributed equally to the work

Experimental details

Single-molecule fluorescence excitation spectroscopy – instrumentation

The two essential requirements to realize room temperature single molecule excitation microscopy are an excitation source that delivers high power, collimated and monochromatic excitation light over a broad wavelength range (>100nm), and the ability to locate and analyze the single molecules. The latter requirement dictates a microscopy approach that combines lateral resolution with the highest sensitivity in detection and smallest possible chromatic aberrations in the excitation branch of the setup.

Full setup description

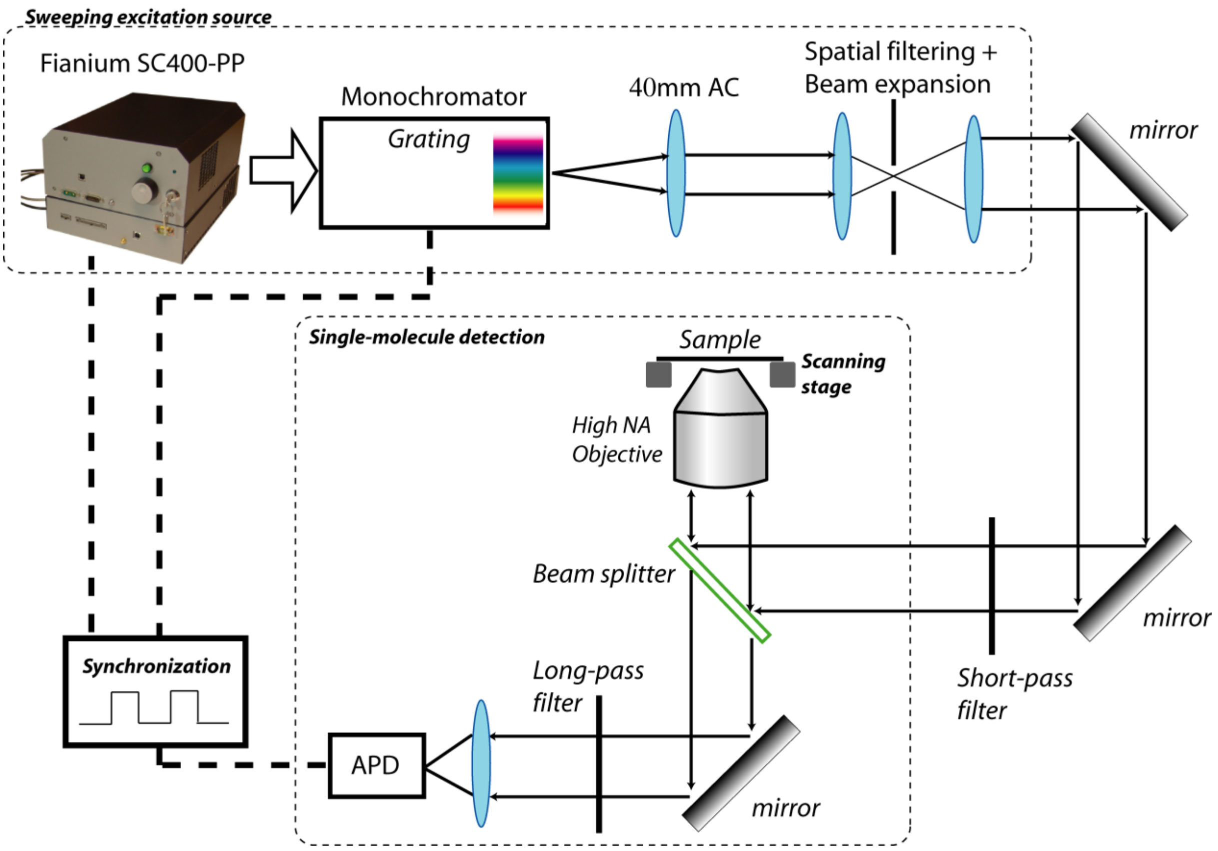

Figure S1: Schematic of the single molecule excitation setup.

Excitation

We used a Fianium SC400-PP pulsed supercontinuum laser source to provide 2W broad spectral excitation power ranging from 400 nm to 2000 nm at 20 MHz repetition rate. The white light was

focused on the entrance slit of an Acton SpectraPro-300i grating monochromator (300 grooves/mm, blazed for 500 nm), light of a very narrow spectral range (<3nm) was collected at the exit slit of the monochromator. To increase the beam quality and to expand the beam size we used a spatial filtering system consisting of achromatic lenses and a pinhole. Wavelength selection was achieved by computer-controlled rotation of the grating inside the monochromator. In this way, excitation sweeps from 520 nm to 620 nm excitation wavelength with step size of 1 nm were achieved. The available excitation power increased with increasing wavelength and was corrected for in the data analysis (see below).

Single-molecule sensitive microscopy

The single-molecule sensitive microscope consisted of a classical, custom built scanning stage confocal microscope. In short, a Zeiss microscope body (Zeiss Axiovert 135 TV, Zeiss, Germany) was equipped with a nano-positioning (PI 527.3CD, Physik Instrumente, Karlsruhe, Germany) stage to scan the sampling plane. A 100x oil immersion objective with an NA of 1.3 was used (Plan-Neofluar 100x/1.30 Oil, Zeiss, Jena, Germany) to focus the excitation light and to collect the emission. A glass plate acted as a beam splitter. The emitted light was collected by the objective, passed through the beam splitter and focused on an avalanche photodiode (APD) (SPCM-AQR-13, Perkin Elmer, USA) with single photon detection efficiency.

To effectively prevent excitation light reaching the detector we used a 620 nm short-pass filter (SP01-633RU-25, Semrock, US) in the excitation path, and in the detection path a 625 nm long-pass filter (LP02-633RU-25) in combination with a 650 nm band-pass filter (FF01-670/30-25).

Synchronization of excitation and detection

In the current experiment we used excitation wavelengths starting from 520 nm up to 620 nm with increments of 1 nm. Per excitation wavelength we recorded the emission for 400 ms, during which we not only recorded the total number of detected photons, but also the evolution of emission intensity within these 400 ms time increments. After 400 ms recording for one excitation wavelength the hardware was preparing for the next wavelength and the detector was disabled giving absolute zero values in the total time-trace. These flags were later used in the data analysis to synchronize excitation wavelength and the measured time-trace offline (see Figure S2).

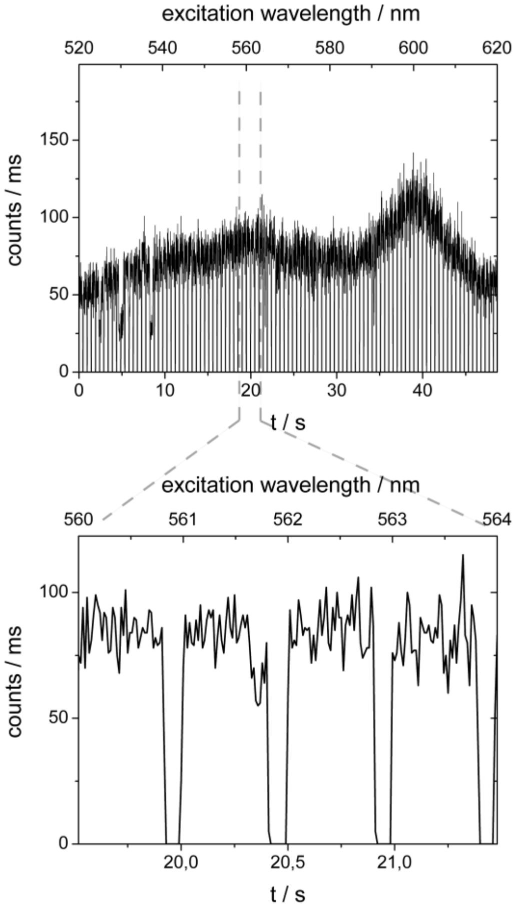

Figure S2: Total time-trace of a single quantum dot, bin time of 5 ms, before any corrections for excitation intensity and detection efficiency were applied. The absolute zero values indicate the change of excitation wavelength where the excitation source and the detector were disabled.

Sample preparation

Sample preparation followed standard protocols for single molecule studies. A highly diluted solution (~0.5 nM) of quantum dots (eFluor650, eBioScience, UK), in 1 % wt PVA in spectroscopically clean water, was spin-cast onto a clean standard microscopy cover glass to immobilize the quantum dots in a thin film of PVA.

Single quantum dot excitation spectroscopy

The experiments were carried out in two steps. First, the single quantum dots were localized by raster scanning an area of the sample using a fixed excitation wavelength and creating an emission intensity image. After the localization, the single quantum dots were positioned in the laser focus and the

excitation wavelength was swept from 520 nm to 620 nm in increments of 1 nm. In a first step to construct the photoluminescence excitation spectrum, the total emission counts per excitation wavelength were integrated (Figure S3).

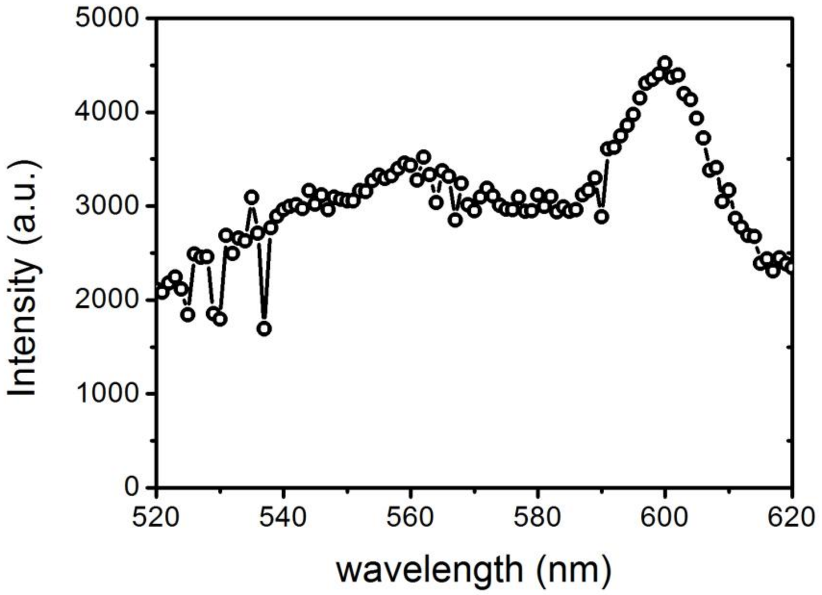

Figure S3: The raw single quantum dot photoluminescence excitation spectrum was calculated by integration of all photons detected per each excitation wavelength. The pronounced 1S(e)-2$S_{3/2}$(h) transition is already clearly visible without further correction of this raw data and was found at the expected wavelength.

Compensating variations in excitation and detection efficiencies

To obtain the photoluminescence excitation spectrum from the raw data as shown in Figure S3, wavelength dependent variations in the excitation power and efficiency as well as in the detection efficiency need to be compensated. This can be achieved by comparing a bulk photoluminescence excitation spectrum obtained with our setup to a photoluminescence excitation spectrum recorded using a calibrated, reference spectrometer. Generally, any fluorophore absorbing and emitting in the analyzed wavelength range can be used for this purpose, but for simplicity we used the QD650 quantum dots that we analyzed in our study.

We recorded the photoluminescence excitation spectrum from a thin film of QD650 quantum dots and a background spectrum using an empty glass cover slip without sample using our photoluminescence excitation setup. Both spectra were recorded using exactly the same settings as for the rest of our analysis, and special care was paid to the presence and avoidance of additional unwanted effects

such as interferences that might arise at certain wavelengths. Further we used a commercial spectrometer (Varian Eclipse) to record the photoluminescence excitation spectrum of the quantum dots used. The correction file was then calculated by dividing the background corrected spectrum recorded for QD650 on our setup by the spectrum recorded using the Varian Eclipse.

$$C(\lambda) = \frac{S(\lambda) - B(\lambda)}{V(\lambda)}$$

Here, *S(λ)* is the recorded excitation spectrum using our setup, *B(λ)* the background spectrum, *V(λ)* the excitation spectrum recorded using the Varian Eclipse and C*(λ)* is the derived correction spectrum compensating for all differences in the excitation and detection efficiency.

To obtain the corrected photoluminescence excitation spectrum of a single emitter from the raw data as presented in Figure S3, the data was background corrected and then divided by the correction spectrum. The resulting corrected spectrum is shown in Figure S4.

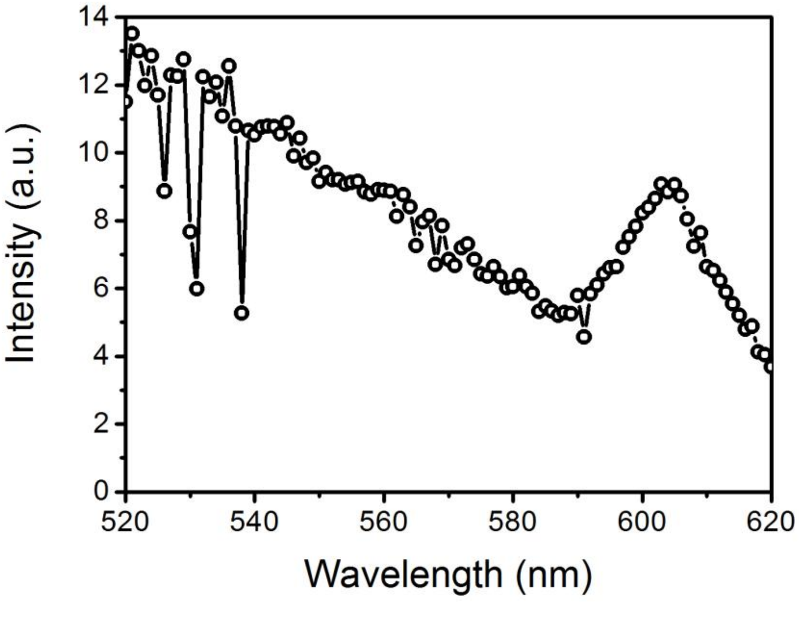

Figure S4: Corrected single quantum dot excitation spectrum.
